# Trajectory Optimization in Terms of Energy and Performance of an Industrial Robot in the Manufacturing Industry

**DOI:** 10.3390/s22197538

**Published:** 2022-10-05

**Authors:** Carlos Garriz, Rosario Domingo

**Affiliations:** 1 Department of Construction and Manufacturing Engineering, Universidad Nacional de Educación a Distancia (UNED), 28040 Madrid, Spain; 2 Body Shop, Volkswagen Navarra, 31170 Arazuri, Spain

**Keywords:** manipulator robot, Kalman method, trajectory optimization, manipulability, electrical energy, simulation

## Abstract

Currently, the high demand for new products in the automotive sector requires large investments in factories. The automotive industry is characterized by high automatization, largely achieved by manipulator robots capable of multitasking. This work presents a method for the optimization of trajectories in robots with six degrees of freedom and a spherical wrist. The optimization of trajectories is based on the maximization of manipulability and the minimization of electrical energy. For this purpose, it is necessary to take into account the kinematics and dynamics of the manipulator in order to integrate an algorithm for calculation. The algorithm is based on the Kalman method. This algorithm was implemented in a simulation of the trajectories of a serial industrial robot, in which the robot has a sealer gun located on its sixth axis and the quality of the sealer application depends directly on the orientation of the gun. During the optimization of the trajectory, the application of the sealer must be guaranteed. This method was also applied to three different trajectories in the automotive sector. The obtained results for manipulability and electrical energy consumption prove the efficiency of the algorithm. Therefore, this method searches for the optimal solution within the limits of the manipulator and maintains the orientation of the final effector. This can be used for a known trajectory.

## 1. Introduction

### 1.1. Research Motivation

The high demand for new and different products requires efficient, flexible and sustainable manufacturing systems. Frequently, efficiency has been sought through automation with machines capable of multitasking that tend to increase energy consumption. This automatization through robots could increase even more in Industry 4.0 environments, where robots, including collaborative robots, emerge and could be more dominant in the production process [1]; in these contexts, robotic integration can lead to higher levels of performance, reliability, sustainability, accuracy and protection from hazardous environments, as well as enable more rational decision making [2]. All of this is common in the automotive sector, where simulation allows verifying multiobjective situations and consequently decreases risks in decision-making [3]. The place where the future vehicle body takes its final shape, called the body shop, is the most automated area. In the workshop, the different vehicle sets and subsets are assembled. This assembly is carried out through different joining techniques that have to achieve the correct vehicle geometry as well as to meet technical design requirements. Given the enormous complexity of these workshops, in order to ensure the correct assembly, the automation level of these shops covers almost all tasks [4], and a very important part is played by manipulator robots.

One of the main tasks that manipulator robots have in the workshop is the application of a sealer to different parts of the vehicle. Sealing is a technique that the designer frequently uses. The sealer is used in assembly processes for its great efficiency, ease of implementation and low cost [5]. In addition, the sealers used in the automotive sector have different purposes, such as ensuring the joining of parts, preventing water entry or avoiding vibrations between parts [6]. The application of the sealer using robots is used widely in this sector since it eliminates the human factor from the process [7]. To ensure an optimal process, it is necessary to use programming adapted to the bond quality of all relevant parameters, such as the flow rate of the sealer, the application speed and the application pressure. In addition, since the sealer application is performed by an industrial robot, the performance of the manipulator robot’s arms must be optimized to guarantee high productivity.

In order to obtain high productivity levels, the trajectories of the robot must be planned correctly. This is usually carried out through the optimization of a goal function defined by the user. In this article, a multigoal function based on the maximization of the performance of the robot and the minimization of the electrical energy for a given trajectory is considered.

### 1.2. Literature Review

The performance index of a manipulator can be understood as a scalar quantity that allows the performance of a robot to be evaluated based on a defined criterion. This performance index can be measured using different criteria, depending on what needs to be evaluated in the robot. The most common indices are kinetostatic and dynamic performance indices. These are defined based on the Jacobian matrix. The work of Yoshikawa [8] is a classic reference on the index of manipulability, and it has been supported by numerous studies. The index is defined as a scalar magnitude capable of measuring the behavior of a robot according to established criteria. Since its introduction, many researchers have presented different criteria for their calculation. For example, Elkady et al. [9] suggested a new algorithm for the calculation of the index of manipulability. This algorithm was called the “Method of decomposition of the singular value”, and it suggests dividing the Jacobian matrix into three submatrices, of which two are orthogonal. The obtained results of the simulation eliminate singular values. In addition, the algorithm establishes a relation between the minimum range of the Jacobian matrix and the matrix of variances. The Jacobian matrix has been similarly employed by different authors for the calculation of the index of manipulability. Doty [10] used the ellipsoids of manipulability and an alternative formulation for the calculation. This work was conducted by defining the space vectors, and it suggested the development of a new formulation based on physical bases. From et al. [11] considered the calculation of manipulability from the restricted Jacobian matrix. This matrix consists of an analytic mapping between the final effector and the joint speeds. It always takes into account the restrictions of the kinematic chain of the manipulator. This approach allows comparing the final effector in non-restricted serial manipulators with others that present certain restrictions. Likewise, Pozna et al. [12] presented a heuristic solution for the problem of inverse kinematics. This one consists of combining the distance between the current position and the desired position of the final effector with the direction leading to better manipulability of the robot. Jin et al. [13] considered the resolution of the singularity problem through the maximization of manipulability. These authors tackled the calculation of real-time manipulability through the establishment of a dynamic neuronal network. This increased the values of manipulability by percentages that were nearly 40% of the average. Dufour et al. [14] proposed the concept of the relaxation of the trajectory, referring to the relaxation of the hard restriction of following the desired trajectory. With this approach, the robot can be diverted from that trajectory. The main advantages are the improvement of the robustness of the manipulator and the maximization of its manipulability.

These performance indices indicate the overall capacity of the manipulation of a robot. This allows the analysis of some fundamental aspects of these tasks. In industry, it is important to optimize the time that the robot takes in a certain trajectory; in this regard, Choi et al. [15] described a computational method to find optimal solutions in terms of route times. They suggested solving the dynamic equation with elemental operations. In addition, this article suggested that with a more precise approach, the suboptimal solution will converge to the real solution of the optimal time. Saravanan et al. [16] suggested minimizing the route time of the trajectories for the manipulator through two evolutionary techniques: the genetic algorithm and differential evolution. These authors concluded that the algorithm using the technique of differential evolution converges more quickly than the one implemented with the genetic algorithm. In addition, better results have been obtained using the technique of differential evolution. Abu-Dakka et al. [17] suggested a genetic algorithm with parallel populations to minimize the route time. The goal of this algorithm is based on building smooth unions using cubic polynomials.

Based on performance indices, another relevant aspect to study is electric energy consumption. The optimization of this parameter has been the subject of numerous analyses. Firstly, Saidur [18] carried out a literature review of the different types of energy losses that take place in a drive motor. These are the main electrical components of an engine. The possible ways of minimizing those losses were explored and identified. An economic analysis was also carried out, identifying the payback period of the different strategies for energy saving. Paes et al. [19] presented systematics for obtaining the trajectory of the optimal energy of an industrial robot. Here, the identification of the parameters to optimize was carried out using a three-phase analyzer of networks. The obtained data were compiled and compared with a model of a dynamic robot parameterized in an optimization routine. This resulted in the specification of a parametric model of the dynamic robot. This was achieved using this model as a dynamic restriction for the problem of the predictive control of the model. The results showed significant improvements in time and energy. Meike et al. [20] summarized several methods for the efficient use of energy. Different approaches and possibilities of energy saving were evaluated. Among these methods, they considered the optimal choice of the robot, methods for intelligent management, the exchange of energy between the controllers of the robot or the establishment of a particular way to wait. Wei et al. [21] analyzed the energy consumption of laser welding in galvanized steel in manufacturing industries and proposed a mathematical model to obtain maximum energy efficiency. This technique achieved energy-saving results of 16%. All of these conclusions allow new energy-efficient processes to be formulated as far as ecological manufacturing is concerned. Another goal of this article is the optimization of electrical energy. Wigström et al. [22] considered a method for the optimization of a manipulator based on the dynamic programming of the existing trajectories. This new method allows solving the optimization problem for a range of operating times for each individual operation. This was only based on a simulation.

All of the studies considered above used a single criterion as the optimization parameter. This can affect other equally important parameters in the planning of robot trajectories, such as the orientation and position of the final effector. The correct orientation and position are necessary to obtain a good application of the sealer. Because of that, it is important to have techniques for multicriteria optimization in order to guarantee the correct optimization of the trajectory without the rest of the parameters being affected. 

It is possible to find studies that developed multigoal methods for serial manipulators. Riazi et al. [23] considered an optimization procedure that reduces energy consumption by as much as 30% and the maximum power of an industrial robot by 60%. In addition, this research presents technologies for reducing electrical energy for a factory. Among the main innovations is the creation of a focused simulation neighborhood for the simulation of the energy flow. These optimization processes are designed for energy production and subsequent automated robot code calculations with different methods to optimize environmental aspects. Paryanto et al. [24] presented an experimental study to validate a dynamic model of an industrial robot to analyze its electric energy consumption. They analyzed and optimized criteria such as the dynamic behavior, cycle time and operating parameters of the robot, among which they highlighted the actual load and its speed. Here, it was observed that the operating parameters were highly influential on the electric energy consumption of the robot. Björkenstam et al. [25] presented an algorithm for efficient generation and movement free from collisions using path planning. In order to discover reasonable compensation among the efficiency and the error, this algorithm suggested the use of a discrete approach of order two. A combination was applied between the torsion of the control element and the cycle time. For this purpose, these authors used an approach based on Gauss–Legendre points, which is equivalent to the midpoint method, whereas Rubio et al. [26] presented an algorithm that solves the optimization problem to find the minimum time trajectory. The planning of the trajectory was related to the parameters of the couple, power and electric energy consumption. The relationship between these parameters allows the user to choose the most efficient way to work according to the parameter that is more interesting. Therefore, this work analyzed the impact of the couple, power and restrictions of electric energy consumption in the generation of minimum time free of collisions.

Mukund-Nilakantan et al. [27] proposed the use of the particle swarm method to achieve a dual objective: the minimization of the cycle time and the optimization of the total electric energy consumption in a packaging line. In other words, they presented a dual approach. Depending on the priority or weight of the parameter, the achieved result varies. In this model, it was observed that the calculation of time based on the cycle time was less compared with the time required for the energy calculation. Zhou et al. [28] used the Taboo-enhanced Particle Swarm Optimization (TEPSO) algorithm, which was developed to solve the multiobjective problem. This algorithm, in which the minimization of electric energy is integrated with the criterion of task execution, uses a multiobjective problem applied to an automated line. The effectiveness of the algorithm was verified by the high local search capacity and the greater search speed relative to other processes. Zhang et al. [29] proposed a non-linear mixed-integer method to minimize electric energy, noise and cycle time on a robotic line. This multiobjective method is based on *Gray Wolf Hybrid* Pareto optimization, which solves the balance of multiple objectives. Li et al. [30] developed a restarted simulated annealing algorithm to address energy optimization and decreased cycle time on an automated assembly line. This algorithm uses a local search with three singularity structures and a restart phase based on a distance allocation procedure. With this, an optimal set is obtained. This algorithm outperforms the genetic algorithm.

As can be observed, it is important to have multicriteria methods because other factors can be negatively affected, such as the orientation of the final effector. Likewise, it is decided to optimize the electric energy of the robot because once the robot is in service, the electric energy is the main economic expense. All of these studies used stochastic techniques to solve their respective goal functions, that is, the characterization of a succession of random variables that evolve over time. Generally, these processes are characterized by making certain assumptions, such as linearity or differentiability. However, in this article, the optimization problem is considered a process involving testing and subsequent correction. This method provides all of the advantages of stochastic methods, such as the ease of implementation or low computational requirements, without needing strong assumptions. This process of testing and subsequent correction is based on the Kalman method, filter or algorithm [31]. This method presents the main advantage that not too many design parameters are necessary. Similarly, the use of this method is also possible even though the goal function cannot be expressed analytically. Overall, the studied and summarized literature suggests that this algorithm can be used in practice for mobile robots [32], especially those orientated toward location tasks, the evasion of obstacles, navigation, surveillance, the follow-up of goals, robotic manipulation or applications of autonomous mobile robots [33].

However, in recent years, with the introduction of the electric car to the market, studies have increasingly focused on batteries and the estimation of their charge. The Kalman filter, being a predictive model, plays an important role. Therefore, this filter is applied to meet these needs. As a result, we have methods such as Wang et al.’s approach [34], in which the charge of lithium-ion batteries is estimated. It also tracks the output voltage. To do this, it uses the extended Kalman filter for real-time estimation. Moreover, the Kalman method was proposed for tracking lithium-ion batteries [35]. This method relates the electromotive energy and the state of charge, optimizing both factors. The results of this article demonstrate that the application of the Kalman filter is effective since the estimation of the load error is less than 2%. Zhou et al.’s method [36] is based on the extended Kalman filter to predict the peak power of battery blocks, depending solely on the representative cells. With this, a multi-parameter method was developed to predict the peak power during a driving program test in electric cars. The results demonstrate, with low complexity and high precision, the robustness of the method. In this field, the Kalman filter can also be combined with other methods to estimate the state of charge of lithium-ion batteries. Chen et al. [37] proposed a method based on a multi-dimensional Taylor network. A multi-step prediction model was built according to the requirements of the Kalman filter. Another field of application of the Kalman filter is related to photovoltaic energy. For example, Motahhir et al. [38] performed a review of the literature on existing algorithms for monitoring maximum power. This article presents the advantages and disadvantages of each method, highlighting the possibility of solving this problem through the extended Kalman filter.

It is from this that an opportunity for research is observed. There is an opportunity to research the Kalman method for a serial manipulator. Therefore, this article is based on a probability density function followed by a Kalman estimator in order to look for the optimal value of the defined goal function. This estimator is capable of updating this density function through the testing process. The continuous iteration of this process allows solutions of high quality to be obtained.

The implementation of the trajectory of a serial robot through the Kalman method is calculated following the minimization of the cost function. This function combines the maximization of the performance of the manipulator and the minimization of electrical energy. The combination of the two factors decreases the energetic cost because the obtained trajectories are softer and far from possible singular points. All of this is considered for three different trajectories in the automotive sector, in which the serial robot must apply a sealer to the body of the vehicle.

Furthermore, the environmental impact of the processes must be taken into account and should be maximized to consider when to optimize electric energy. Generally, the environmental impact of the automotive industry has received limited attention. Keep in mind that industrial manufacturing is the largest sector in terms of energy demand and greenhouse emissions (more than 30% of the total). Consequently, the need to mitigate the environmental impact of manufacturing processes makes energy efficiency a key factor in sustainable production. Therefore, Renna and Materi [39] performed an exhaustive analysis of the methods and tools aimed at improving energy efficiency. For this, they carried out a thorough review of the literature, this being the starting point for numerous investigations. Production processes are important components responsible for the environmental impact [40], although they are still poorly documented at the environmental level. Shrouf et al. [41] proposed a method to guarantee ecological manufacturing through multi-level awareness. This multi-level awareness is achieved by integrating production data at the operational level. Through a specific study, the authors managed to integrate data on electrical energy and production to achieve the multi-level awareness of the energy used in production. Mukund-Nilakantan et al. [42] developed an algorithm to minimize the carbon footprint of production lines. To do this, they proposed a coevolutionary multiobjective algorithm. This algorithm achieves a strong local search capacity, and it is faster than others. This algorithm was compared with other multiobjective functions, demonstrating the effectiveness of the proposed model. It is important to consider the environmental impact in the early stages of product and process development. Borsato [43] created an information model using building blocks, process parameters, 3D characteristics and thermodynamic analysis. The model was implemented, and the results show that it is feasible to correlate all data through semantic relationships and thus estimate the indicators related to energy efficiency. Therefore, this article presents the means to access energy efficiency from an integrated system, opening the possibility of extending the model to other manufacturing processes. Rodrigues-Vaz et al. [44] analyzed the environmental impact and innovation in the car industry; they found that although there is high environmental innovation, there is also a need for empirical studies in automotive companies on environmental practices and their impact on innovation.

### 1.3. Research Objectives

Numerous investigations have been conducted in which many path-planning methods focus on minimizing time, minimizing the displacement of joints [45] or minimizing electrical energy consumption. Those that adopt the criterion of minimizing electrical energy consumption as part of a multiobjective problem have used stochastic methods. Among the most frequently used methods are genetic algorithms, the Lagrangian technique and tunnel algorithms.

However, there is no evidence of trajectory-planning developments involving a multiobjective method using the Kalman method. 

For the reasons described above and as a novelty in the literature, this article is formulated with a dual objective: to obtain the maximum performance of a manipulator robot and to minimize the consumption of electrical energy in economic and environmental terms. This was performed for given trajectories using the Kalman method, which, by means of testing and correction, provides a solution for a shorter calculation time than other stochastic algorithms and reduces the effects of unwanted inputs. A research opportunity is the use of a probability density function followed by a Kalman estimator in order to look for the optimal value of the defined goal function. As seen later, the results and calculations can be extrapolated to any serial manipulator and any trajectory. Therefore, the suitability of the method was tested.

## 2. Methodology

This section is structured in four subsections, first devoted to the scheme of the process, followed by sustainable trajectory optimization and the study of the kinematics and dynamics of a robot with six degrees of freedom (DOFs) and a spherical wrist. The use of the Kalman algorithm as the method of optimization is then demonstrated.

### 2.1. Flowchart for the Optimization of Trajectories

**Figure 1:**
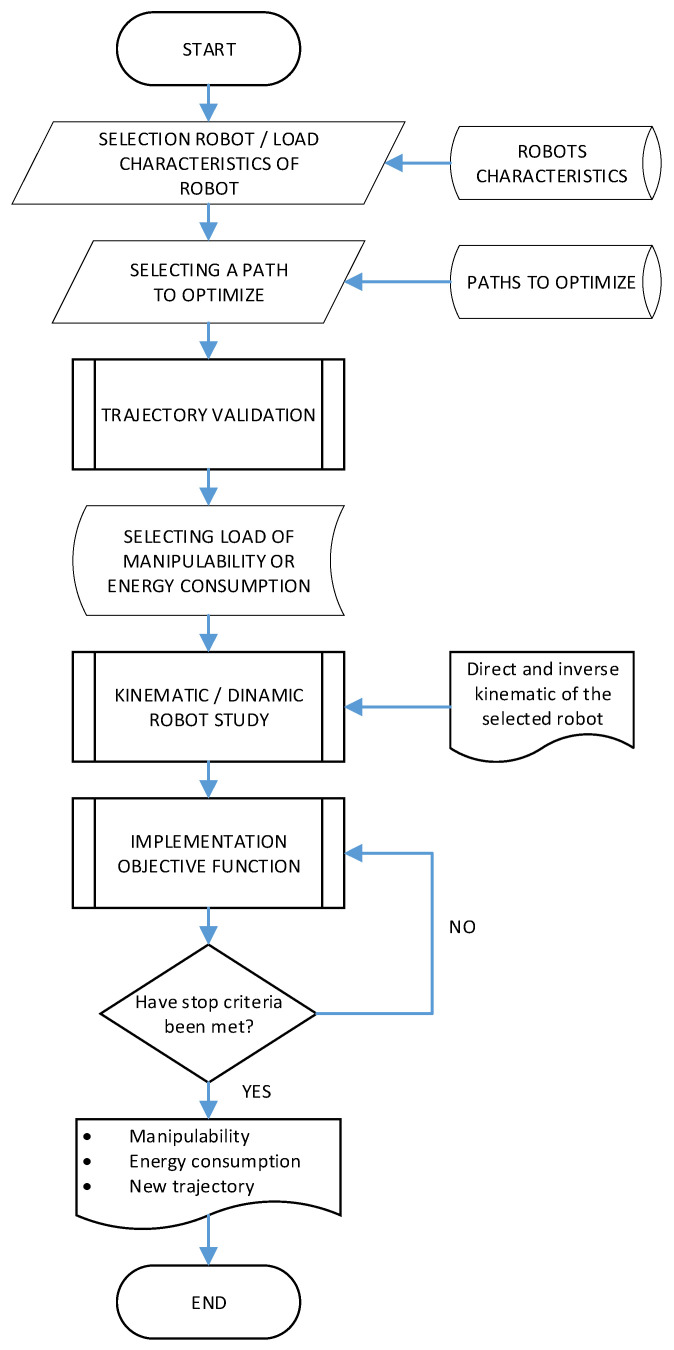
Flowchart followed for trajectory of sustainable optimization.

The flowchart shown in Figure 1 represents the sequence for the optimization of trajectories in any manipulator. This corresponds to specific created software in which the manipulator and trajectory to study are selected. With that, the software optimizes the performance and the electric energy of the manipulator. Notice that to obtain the corresponding results, it is necessary to study the kinematics and dynamics of the selected manipulator. Later, the objective function defined to select the weight for each of the optimization criteria (manipulability and electric energy) is implemented. Once the iterative algorithm obtains the results complying with the given unemployment conditions, it shows the results of both optimization criteria.

### 2.2. Study of the Kinematics of a Robot with Six DOFs and a Spherical Wrist

**Figure 2:**
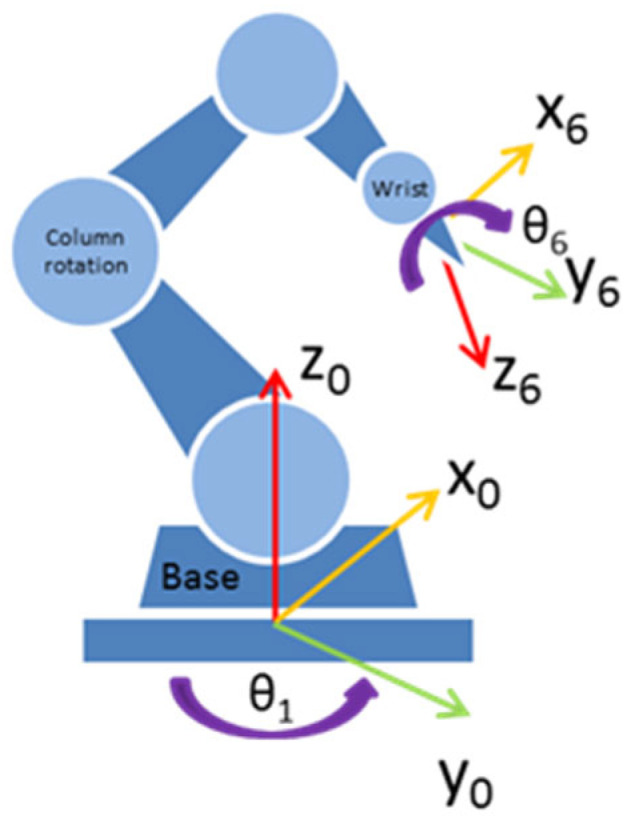
Manipulator robot with a sealer gun.

Since most of the industrial robots used in the automotive sector are serial manipulators with six degrees of freedom and spherical wrist, these factors are taken as an example to apply the proposed optimization method. For this simulation, the KUKA KR30-3 robot was chosen, which is a robot in which a gun for sealer application is attached to the final effector. The components and the movements of the robot are shown in Figure 2.

#### 2.2.1. Direct and Inverse Kinematics of Serial Manipulator with Six DOFs and Spherical Wrist

Direct kinematics refers to the position and orientation of the final effector. It uses the Denativ–Hartenberg convention. In addition, the same convention is used to find the inverse kinematics of the robot. In this case, the inverse kinematics can be solved by geometrical methods [46]. It is solved in two steps because, in manipulators with spherical wrists, the movement of the three last links does not change the position of the center of the manipulator’s wrist. The orientation and position of the end of the gun with regard to the base of the robot are known. Hence,

(1) P.wrist40=T60×P.wrist46,

where $P.wrist_{4}^{0}$ is the position of the wrist relative to the base of the robot, and $P.wrist_{4}^{6}$ is the position of the end of the tool relative to the wrist. 

#### 2.2.2. Differential Kinematics of Serial Manipulator with Six DOFs and Spherical Wrist

After this, the Jacobian matrix must be used. The differential kinematics is treated with regard to the center of the wrist.

(2) $$V_{w}=\left[\begin{array}{c} w_{w}\\ \dot{p_{w}} \end{array}\right]=J\left(\theta \right)\times \dot{\theta },\;$$

where *w_w_* is the angular speed of the wrist, and *p_w_* is the linear speed of the wrist.

(3) $$w_{m}=J_{ra}\times {\dot{\theta }}_{a}+J_{rw}\times \dot{\theta_{w}};\;{\dot{\theta }}_{w}=J_{rw}^{-1}\times \left(w_{w}-J_{ra}\times {\dot{\theta }}_{a}\right),$$

(4) $$\dot{p_{w}}=J_{ta}\times {\dot{\theta }}_{a};\;{\dot{\theta }}_{a}=J_{ta}^{-1}\times \dot{p_{w}}\;,$$

Analogously, a similar process is followed for the case of acceleration.

#### 2.2.3. Manipulability of a Serial Manipulator with Six DOFs and a Spherical Wrist

**Figure 3:**
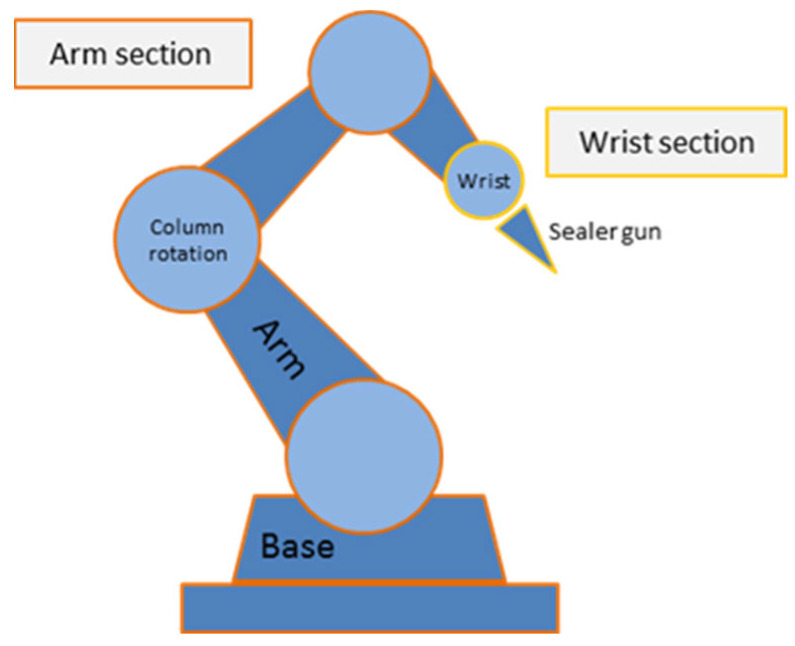
Robot manipulator decomposition (adapted from Yoshikawa [47]).

With the kinematics of the manipulator already known, the manipulability can be obtained. This is one of two parameters to optimize in this article. The calculation of manipulability is based on the studies carried out by Yoshikawa [8]. Yoshikawa argued that manipulability can be reflected as an ellipsoid in Euclidean space. In addition, for the calculation of manipulability, the decomposition of the manipulator proposed by Yoshikawa [47] is used. The authors propose decomposition into two sections for a manipulator with six degrees of freedom and a spherical wrist (see Figure 3). 

With the described method, the Jacobian matrix of a manipulator with six degrees of freedom and a spherical wrist is given by

(5) $$J=\left[J_{a1}\;\;\;\;J_{a2}\;\;\;\;J_{a3}\;\;\;\;J_{w4}\;\;\;\;J_{w5}\;\;\;\;J_{w6}\right],$$

where $J_{ai}$ is the Jacobian matrix of the *i*th articulation of the arm (for i = 1, 2, 3), and $J_{wi}$ is the Jacobian matrix of the *i*th articulation of the wrist (for i = 1, 2, 3).

So, the following Jacobian matrix is obtained:

(6) $$J=\left[\begin{array}{c} z_{1}\;x\;\;p_{1}\\ z_{1} \end{array}\begin{array}{c} \;\;\;\;z_{2}\;x\;\;p_{2}\\ \;\;\;\;z_{2} \end{array}\begin{array}{c} \;\;\;\;z_{3}\;x\;\;p_{3}\\ \;\;\;\;z_{3} \end{array}\begin{array}{c} \;\;\;\;z_{4}\;x\;\;p_{w}\\ \;\;\;\;z_{4} \end{array}\begin{array}{c} \;\;\;\;z_{5}\;x\;\;p_{w}\\ \;\;\;\;z_{5} \end{array}\begin{array}{c} \;\;\;\;z_{6}\;x\;\;p_{w}\\ \;\;\;\;z_{6} \end{array}\right],$$

where *z_i_* is the unit vector in the direction of the *z*-axis, and *x* denotes the vector product.

The index of manipulability is defined as

(7) $$w=\det\sqrt{J\left(q\right)\times J^{T}\left(q\right)}\;,$$

### 2.3. Study of the Dynamics of a Robot with Six DOFs and a Spherical Wrist

Since the aim is to simulate movements and, based on these, optimize the performance of the manipulator and electric energy, a study on the dynamics for the selected manipulator must be carried out: the KUKA KR30-3 robot. For the study of dynamics, the Newton–Euler formulation is adopted for its easy implementation [48]. In addition, this formulation is more computationally efficient [49].

#### 2.3.1. Inverse Dynamics

Focusing on the calculation of inverse dynamics for this manipulator, the Newton–Euler algorithm can be divided into two phases: the first phase is the forward propagation of the velocities and accelerations of the joints themselves. This occurs from articulation 1 to 6 of the manipulator under study. Moreover, the second phase of calculation is the backward propagation (from articulation 6 to 1) of the resulting pairs and forces. Since the joints present in this manipulator are rotational, the equations of the first phase of the algorithm are presented below.

Propagation of angular velocity:

(8) $$w_{i}=w_{i-1}+z_{i-1}{\dot{\theta }}_{i},$$

Propagation of angular acceleration:

(9) $$\dot{w_{i}}={\dot{w}}_{i-1}+z_{i-1}\ddot{\theta_{i}}+w_{i-1}x\;z_{i-1\;}{\dot{\theta }}_{i}\;,$$

Propagation of linear velocity:

(10) $$v_{i}=v_{i-1}+w_{i-1}\;x\;r_{i}\;,$$

Propagation of linear acceleration:

(11) $${\dot{v}}_{i}={\dot{v}}_{i-1}+{\dot{w}}_{i-1}\;x\;r_{i}+w_{i}\;x\;\left(w_{i}\;x\;r_{i}\right)\;,$$

To be able to add accelerations and speeds, they must be in the same frame of reference *i*. Therefore, they are presented in reference system *i*.

Propagation of angular velocity:

(12) wii=Rii−1(wi−1i−1+zi−1i−1θ˙i),

Propagation of angular acceleration:

(13) wi˙i=Rii−1(w˙i−1i−1+zi−1i−1θi¨+wi−1i−1x zi−1i−1θ˙i) ,

Propagation of linear velocity:

(14) vii=Rii−1(vi−1i−1+wii−1  x rii) ,

Propagation of linear acceleration:

(15) v˙ii=Rii−1(v˙i−1i−1+w˙ii−1 x rii+wii x (wii x rii)) ,

Linear acceleration of the center of mass:

(16) v˙ici=(v˙ii+w˙ii x rici+wii x (wii x rici)) ,

where *r_i_* is the position of system *i* with respect to system *i-1*, *r_ci_* is the position vector of the center of mass of link *i* with respect to system *i-1*, *v_i_* is the system speed *i*, *v_ci_* is the linear velocity of the center of mass of link *i*, ${\dot{v}}_{i}$ is the linear acceleration of system *i*, ${\dot{v}}_{ci}$ is the linear acceleration of the center of mass of link *i*, *z_i_* is the *z_i_*-axis with joint coordinate *θ_i+1_*, $w_{i}$ is the angular velocity of link *i*, ${\dot{w}}_{i}$ is the angular acceleration of link *i*, and Rii−1 is the orthonormal rotation matrix that leads from *i-1* to *i*.

The effect of gravity is included by assigning the magnitude of gravity but in the opposite direction to the linear acceleration of the robot base link [50].

#### 2.3.2. Direct Dynamics

For the study of direct dynamics, the general equation for a manipulator with *n* degrees of freedom must be considered.

(17) $$\vec{Q}=M\left(q\right)\ddot{q}+C\left(q,\dot{q}\right)\dot{q}+F\left(\dot{q}\right)+G\left(q\right),$$

where $\vec{Q}$ is the generalized vector of forces, q is the articular coordinates, $\dot{q}$ is the articular velocities, $\ddot{q}$ is the articular accelerations, *M* is the complete inertia matrix of the manipulator, *C* is the Coriolis Effect, *F* is viscous and Coulomb friction, and *G* is gravity.

As can be seen, this recursive algorithm is developed with a forward recursion for the calculation of linear/angular velocities, linear/angular accelerations and their center of mass. Once this is calculated, the backward recursion is performed to find the forces and torques.

#### 2.3.3. Electric Energy of the Manipulator

All existing robots have motors capable of producing movement. The most common are DC motors and servomotors. Industrial robots generally use servomotors that are permanent magnet synchronous motors [51]. The electrical power consumed by the motors can be denoted as

(18) $$P_{Total}=\sum_{i=1}^{6}P_{Ci}+P_{femi}\;,$$

where $P_{Ci}$ is the heat output produced by the Joule effect on each of the engines, and $P_{femi}$ is the power to produce movement in each of the engines. With the previous equation, the electrical energy of the manipulator in a trajectory can be calculated for optimization:

(19) $$E.energy=\sum_{j=1}^{n}P_{Totalj}\times t_{j}\;,$$

where *E. energy* is the electrical energy of the manipulator for a given trajectory and the set of the engines that compose it, and $P_{Totalj}$ is the instantaneous electrical power consumed by the motors at joint j of the trajectory under study.

### 2.4. Method of Optimization: Kalman Algorithm

As explained previously, the Kalman method is used to optimize the manipulator trajectories. Remember that these trajectories are optimized according to two main criteria: manipulability and electric energy. The Kalman method [31] makes use of a probability function and a Kalman estimator [52]. This is an iterative procedure in which there is first a random generator of probability functions that produces a collection of *N* vectors distributed in an array of variances and covariances *Σ(j)*, as well as a vector of means *m(j)* in the form:

(20) $$k\left(j\right)=\left\{k^{1}\left(j\right),k^{2}\left(j\right),\ldots k^{N}\left(j\right)\right\}\;,$$

where *k^i^(j)* is the *i*th vector that generates in iteration *k*.

The random generator is applied to the cost function. The described algorithm modifies the vector of averages and the matrix of variances of the generator until a solution of the desired quality is obtained. A measurement process followed by an estimator is introduced to achieve the desirable estimation of the optimum. For this, the average of the candidates that best represents the optimum is calculated. So, it should be:

(21) $$\xi \left(j\right)=\frac{1}{N_{\xi }}\sum_{i=1}^{N_{\xi }}k^{i}\left(j\right)\;,$$

where *N_ξ_* is the number of the best examples to consider. The measures are given by:

(22) $$\xi \left(j\right)=k_{optimo}+\upsilon \left(j\right)\;,$$

where *υ(j)* is an unknown disturbance. This uncertainty is estimated, taking into account the information available. The lack of knowledge of the optimum is taken into account using the vector of variances associated with the best samples. Therefore,

(23) $$\upsilon \left(j\right)=\frac{1}{N_{\xi }}{\left[\sum_{i=1}^{N_{\xi }}\left(k_{1}^{i}\left(j\right)-\xi_{1}\left(j\right)\right),\ldots ,\sum_{i=1}^{N_{\xi }}\left(k_{n_{j}}^{i}\left(j\right)-\xi_{n_{j}}\left(j\right)\right)\right]}^{T},$$

In such conditions, a Kalman estimator can be used to perform the estimation. Taking the Kalman equations as a basis [31], the rule to update the Gaussian generator would be:

(24) m(j+1)=m(j)+P(j)(ξ(j)−m(j)),

(25) $$\sum^{\;}\left(j+1\right)=\left(I-b\left(j\right)\times P\left(j\right)\right)\times \sum^{\;}\left(j\right)\;,$$

(26) $$P\left(j\right)=\sum^{\;}\left(j\right){\left(\sum^{\;}\left(j\right)+d\left(j\right)\right)}^{-1},$$

where *d(j)* is a diagonal matrix with a diagonal corresponding to the vector of variances υ(j), and *b(j)* is a coefficient used to decrease the time of the variance matrix $\sum^{\;}\left(j\right)$.

To initialize and adjust the parameters of the Gaussian generator, it should cover the full search space. For this reason:

(27) $$m_{0}=\left[\begin{array}{c} {µ}_{1}\\ \vdots \\ {µ}_{nk} \end{array}\right],\;\sum_{0}=\left[\begin{array}{ccc} \sigma_{1} & 0 & 0\\ 0 & \ddots & 0\\ 0 & 0 & \sigma_{nk} \end{array}\right]\;,$$

where

(28) $${µ}_{i}=\frac{k_{i\sup+k_{i\;inf}}}{2},\;for\;i=1,\ldots n_{k}\;,$$

(29) $$\sigma_{i}=\frac{k_{i\sup+k_{i\;inf}}}{6},\;for\;i=1,\ldots n_{k}\;,$$

As a result, 99% of the samples are generated in the interval ${µ}_{i}+3\sigma_{i}$.

In summary, the steps that the presented algorithm follows to minimize the cost function are the following:

1. Set the number of points N, the number of best candidates *N_ξ_* and the slowdown coefficient α;
2. Generate the sequence of N vectors for each iteration *j* according to the Gaussian distribution;
3. Perform the measurement process;
4. Update the stop rule;
5. Check the stop rule. If it is not satisfied, go to the Gaussian generator step.

The Kalman algorithm is used to maximize the manipulability and minimize the electric energy of the manipulator. Thus, the cost function is defined as the function that must be minimized.

(30) $$Q\left(q\right)=\frac{E.energy}{P_{n}\times t}\times P.electrical\;energy+\left(1-\frac{w_{m}}{w_{max}}\right)\times P.manip,$$

where *E.energy* is the electrical energy consumption of the trajectory, $P_{n}$ is the nominal power of the manipulator, *t* is the time of the trajectory, *P.electrical energy* is the weight that is assigned to the electrical energy consumption of the trajectory, *P.manip* is the weight that is assigned to the manipulability of the trajectory, $w_{m}$ is the average manipulability of the manipulator, and $w_{max}$ is the maximum manipulability of the manipulator.

This cost function is dependent on the chosen manipulator and, obviously, on the restrictions that are imposed. Therefore, it can be affirmed that the correct cost function is

(31) $$Q{\left(q\right)}_{c}=Q\left(q\right)+P.res\times \sum_{n=1}^{N}\max\left(R_{i}\left(q\right),0\right),$$

where *N* is the number of restrictions imposed (limits on the robot and articular speeds), $R_{i}\left(q\right)$ is the function of the ith restriction, and *P.res* is the weight given to restrictions.

**Figure 4:**
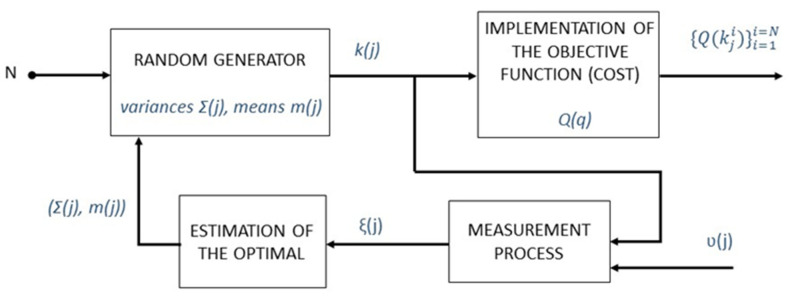
Implementation of the proposed algorithm.

The proposed algorithm is configured based on a binomial variable, for which the best samples are chosen. With this, a rule of unemployment is proposed based on 15 iterations when each of the criteria to optimize is met such that the deviation in manipulability in absolute value between the current iteration and the previous iteration is less than 0.001, and the deviation of electric energy in absolute value between the current iteration and the previous iteration is less than 0.0001. The implementation process of the proposed algorithm can be seen in Figure 4.

There are other multiobjective optimization methods. One of the most popular is the Pareto front [53]. Although this method may present a clear sample of results, it was excluded due to its disadvantages. It has a higher computational cost, different weights may lead to the same solution, and all of the problems have to be converted to the same type. 

## 3. Case Study: Serial Robot

KUKA KR is a family of industrial robots with six degrees of freedom and a spherical wrist, which allows the control of points and trajectories with great precision. Within this family of robots, there is a variety of models depending on the load supported by the tip or 6 axes. The load is not significant because the robot has a sealer gun. Because of that, the robot model KUKA KR30-3 was chosen for the simulation. 

### 3.1. Robot Characteristics

**Figure 5:**
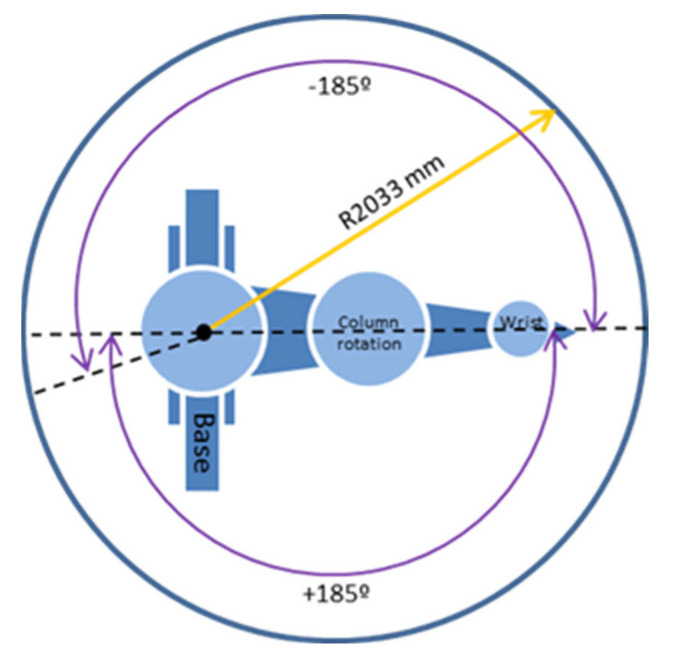
Range of motion of the robot, modified from [55].

The main technical characteristics of the robot are presented below. They include the workspace in which the robot is capable of acting (Figure 5). In addition, it provides a description of its basic characteristics (Table 1). The characteristics of the tool fitted on its end for the sealer gun application are also considered.

As mentioned, the tool fitted in the robot is a sealer gun, model ADK6000, with a multibox 6000 of the SCA brand [54]. This application of the sealer cannot be carried out at room temperature, so it must be heated in the container to ensure its correct application.

**Table 1:** Position and speed of each axis of the robot, modified from [55].

Robot Axis	Position [°]	Speed [°/s]
1	±185	140
2	–35/–135	126
3	–158/–130	140
4	±350	260
5	±119	245
6	±350	322

### 3.2. Trajectories

To evaluate the correct behavior of the method, three trajectories in which the sealer is applied are proposed using the robot described above. These three trajectories were obtained from real trajectories used in the automotive sector. All of these trajectories are given in the body shop.

**Figure 6:**
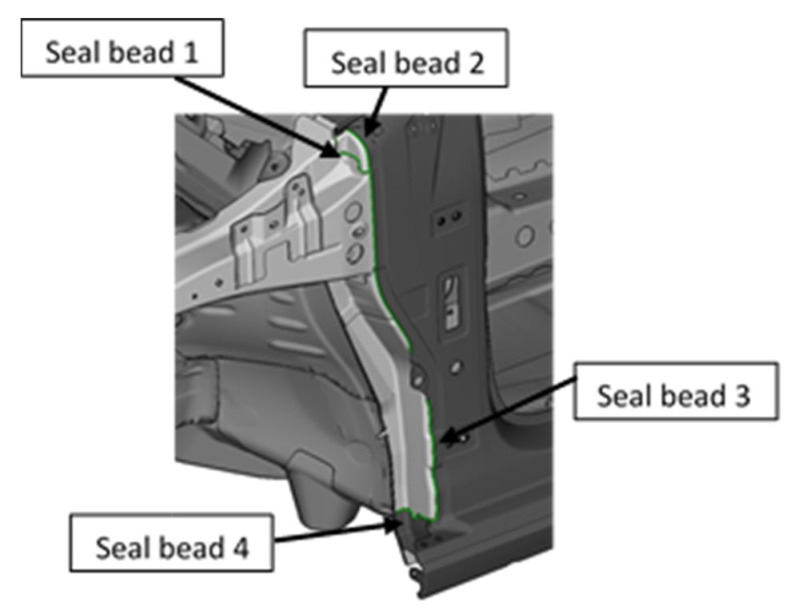
Trajectory of sealer application of Montante A (Trajectory 1).

The first trajectory, called the “montante A” trajectory, is the application of the sealer to the front of the vehicle, which prevents water from entering the cabin. This trajectory has a 71-point program because of the complex geometry of the vehicle (see Figure 6).

**Figure 7:**
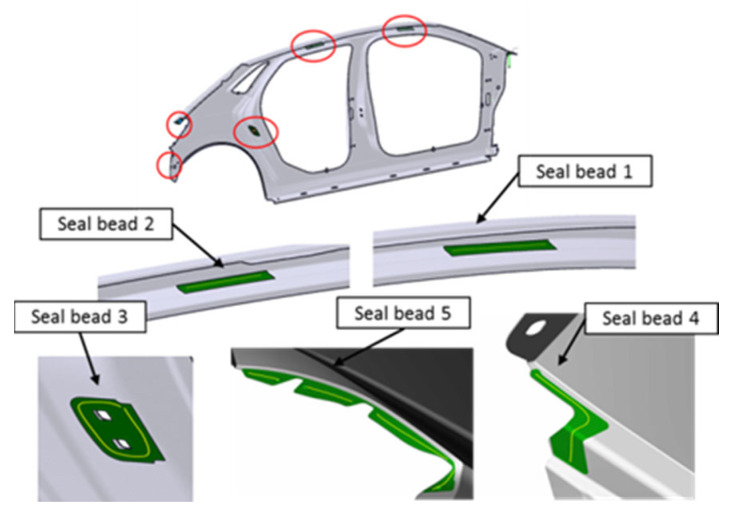
Trajectory of sealer application to the left side of the vehicle (Trajectory 2).

The second trajectory has a 104-point program, and it is the application of the sealer to the left-hand side panel. This sealer has the objective of pasting the part, as we can see in Figure 7, with some pieces that are added in later operations.

**Figure 8:**
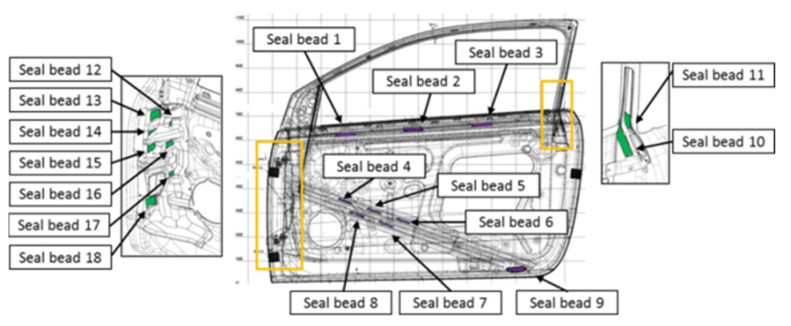
Trajectory of sealer application to the left side door (Trajectory 3).

In addition, the last trajectory is the application of the sealer to the front left-hand side door of the vehicle. Here, the sealer has two different purposes: the sealer is used as a paste to fix two pieces together, and it also prevents noise due to the vibrations of the vehicle. This application has a 135-point program (see Figure 8).

### 3.3. Robot Characteristics

This section presents the obtained results based on the given algorithm. It shows the values of manipulability and electric energy for the three trajectories presented previously.

**Figure 9:**
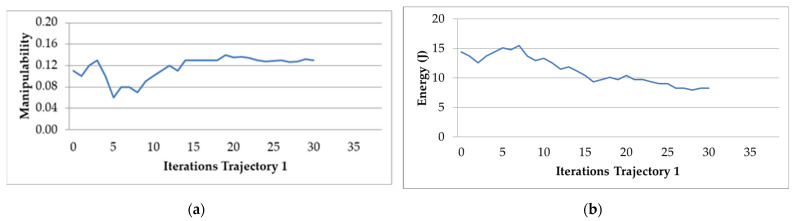
Behavior of the optimization algorithm for Trajectory 1: (**a**) average manipulability; (**b**) average electric energy consumption.

First, to evaluate the cost function as required, it is necessary to know the weights that should be assigned to each of the criteria. For that, for the first trajectory chosen, the algorithm was experimentally applied with different weights to the criteria of manipulability and electric energy. The obtained results are shown in Figure 9a,b.

#### 3.3.1. Weights

As is seen, the cost function to be minimized will depend on the weight assigned to each criterion (manipulability and electric energy). The assigned weight is between 0 and 1. The weight was assigned experimentally based on the application of the algorithm in Trajectory 1. Some of the results are shown in Table 2.

Table 2 shows that the algorithm with a weight of 0.99 for consumption and 0.01 for manipulability has the lowest consumption but also the lowest manipulability. In addition, it requires a greater number of iterations than the rest of the combinations. 

Observing Table 2, assigning a weight of 0.95 for electrical consumption and 0.05 for manipulability data stands out as the most balanced weight solution. It is observed that it presents a similar number of iterations to the rest, it has the second-lowest consumption of all, and it presents the highest manipulability. 

This chosen combination of weights is checked against the rest of the implemented trajectories. 

#### 3.3.2. Manipulability and Electric Energy

In this subsection, the behavior of the parameters under study and how the algorithm optimizes the trajectory in each iteration are shown. The behavior of the optimization algorithm is presented in Figure 9a,b. This algorithm takes into account the manipulability factor and electric energy consumption in Trajectory 1. These graphs show that both factors stabilize in iteration 30.

**Figure 10:**
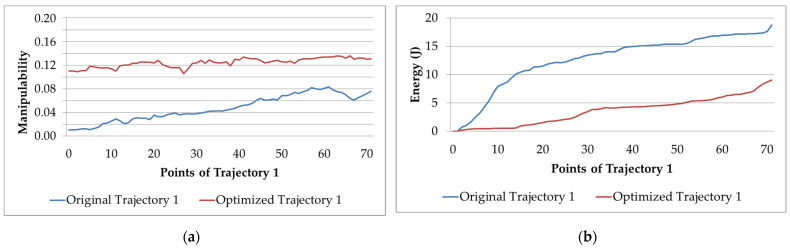
Original and optimized Trajectory 1: (**a**) manipulability; (**b**) electric energy consumption.

Figure 10a,b show the behavior of the manipulability factor and electric energy consumption for the original/optimized Trajectory 1.

Figure 10a shows that the manipulability of the optimized trajectory is greater than that of the original. In addition, the performance of the manipulator increases. It is also appreciated that it remains constant. Moreover, Figure 10b shows that the electric energy of the manipulator decreases after the optimization. 

In trajectories as complex as this, a decrease in the electric energy is especially noticeable. In addition, the manipulator performance improves. This is because the trajectories of the joints of the robot must change.

**Figure 11:**
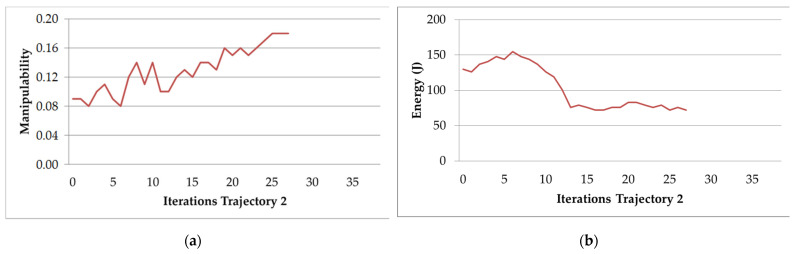
Behavior of the optimization algorithm for th Trajectory 2: (**a**) average manipulability; (**b**) average electric energy consumption.

Figure 11a,b present the behavior of the optimization algorithm for Trajectory 2. In both Figures, it is observed that the optimization algorithm stabilizes in iteration 27. This is the iteration in which the stop criterion is met. In Figure 11a, the manipulability increases to a value of around 0.18 and stabilizes, while in Figure 11b, the electric energy reaches more stable values at iteration 27.

**Figure 12:**
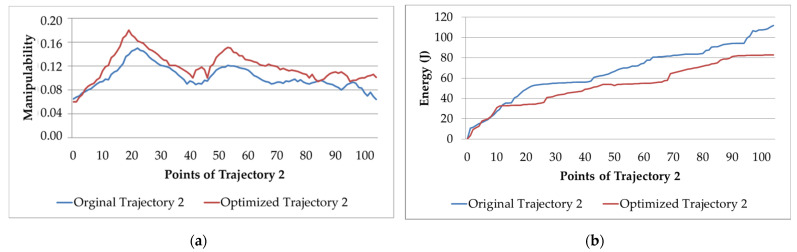
Original and optimized Trajectory 2: (**a**) manipulability; (**b**) electric energy consumption.

Figure 12a,b present the graphs of manipulability and electric energy that are obtained with the original Trajectory 2 with its respective optimized trajectory.

Figure 12a shows that the optimized trajectory manipulability factor largely resembles the original trajectory. Note that at point 3 and point 84, the manipulability of the original one is higher than that of the optimized trajectory. These points are the ends of the sealer beads in a certain plane. Here, the manipulator needs to reorient its joints, and therefore, the manipulability may decrease. In Figure 12b, which shows the electric energy, a situation analogous to the results for the manipulability factor can be observed. Between points 4 and 11, there are points where there is no application of the sealer (only reorientation and displacement to apply the next bead of sealer). Here, the electric energy is lower than that of the original trajectory. The optimization algorithm also achieves a reduction in trajectory time through its optimization. At points where it is not necessary to maintain the orientation and position of the end effector (when applying sealer), it plays with the manipulator’s speed and acceleration.

**Figure 13:**
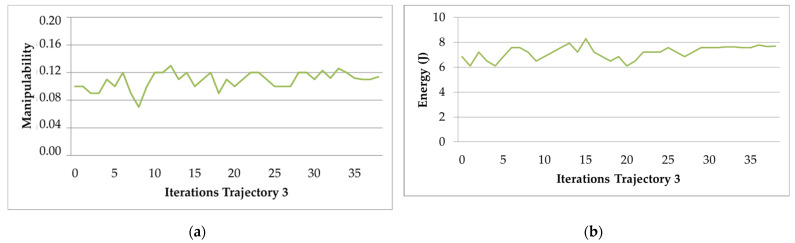
Behavior of the optimization algorithm for Trajectory 3: (**a**) average manipulability; (**b**) average electric energy consumption.

The behavior of the manipulability factor and electric energy optimization algorithm for Trajectory 3 is presented in Figure 13a,b. The algorithm is stabilized in iteration 38, as can be seen in both figures.

**Figure 14:**
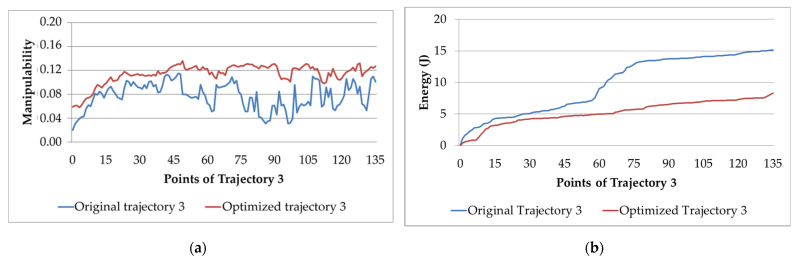
Original and optimized Trajectory 3: (**a**) manipulability; (**b**) electric energy consumption.

Figure 14a,b show the manipulability and electric energy factor of the original Trajectory 3 against its optimized trajectory. In Figure 14a, it can be seen that the manipulability factor is also higher than that of the original trajectory and tends to be more stable. The manipulability in the original trajectory looks more unstable. However, the manipulability in the optimized trajectory is more stable, growing from 0.06 to 0.1–0.14. We recall that Trajectory 3 is the application of sealer beads to the vehicle door. The part dimensions are relatively small, and the number of sealer counts in different large application planes due to constant reorientations to ensure the correct application has a direct impact on the measurement of the manipulator performance. With the implementation of the optimization algorithm, this factor is seen to improve.

Regarding the electric energy, in Figure 14b, it is clearly observed that it is smaller than that in the original trajectory, attenuating the jump in consumption that occurs at point 56 in the original one.

Table 3 shows a summary of all of the studied trajectories. The influence of the optimization algorithm and the best percentages for the parameters to be optimized and trajectory times can be seen. In general, it is clear that all of these factors have a great economic impact. Therefore, Table 4 shows the theoretical increase in production capacity for each of the trajectories, as well as the economic savings involved.

In comparison with other studies, the method used for the optimization of manipulability is effective and precise, and it improves the results relative to other approaches. As clear examples, From et al. [11] considered the calculation of manipulability from the restricted Jacobian matrix for trajectory times of around 50 s (a trajectory similar to Trajectory 1 in this article). From et al. [11] obtained an average improvement of 40% compared with other studies. However, our method achieved improvement percentages higher than 50%.

Regarding the trajectory times, it can be said that this method also improves the optimization percentages compared to other investigations. There are, for example, studies such as the one by Abu-Dakka et al. [17]. These authors suggested the use of a genetic algorithm with parallel populations to minimize the route times. This procedure demonstrated improvements of around 3 s per trajectory. Abu-Dakka et al. [17] considered three types of constraints: the kinematics/dynamics of the robot and the payload constraints. The last constraint is different than that in our article. In this study, it was not considered because it involved a robot with a sealer gun. The robot does not need to move parts.

According to electricity consumption optimization, there are some investigations, such as Riazi et al. [23], in which maximum optimization percentages of 30% were achieved. These percentages were obtained by scaling the speed and acceleration on the existing trajectory. On the contrary, the method proposed in this paper achieves improvement averages percentages of more than 40%, except in cases where the trajectory involves numerous changes in acceleration as a result of large displacements. 

The KPIs (Key Performance Indicators) in the automotive industry are divided according to the type of process applied to the vehicle in its given manufacturing stage. In a factory, there are four main stages (shops): the press shop, body shop, paint shop and assembly. This article presents a study on a robot in a body shop. Here, there are three main indicators of energy (e-KPIs). The first one is the energy intensity [56]. The energy intensity is related to the electrical energy consumption and the number of vehicles. Another indicator is the basic electricity charge indicator. This concept is defined by the difference in the minimum electricity consumption and the maximum electricity consumption when it has produced parts. The calculation is performed daily. The last indicator refers to the energy yield potential. However, this is not presented because it requires a complete thermodynamic study of the assembled part. Since it is not an object of study, it is not taken into account. Note that this article focuses on a robot with a sealer gun.

The results shown in Table 5 clearly indicate that, in the optimized trajectory, the results for energy intensity are better. This is logical because it produces more parts with less consumption. Moreover, the basic electricity charge indicator is also improved. This is because the average electrical consumption is better, so this indicator tends to zero.

## 4. Conclusions

This article presents a multiobjective problem based on the Kalman algorithm. This algorithm simultaneously studies the factors of manipulability and electric energy. This was implemented for a serial robot with six degrees of freedom and a spherical wrist. This robot has a sealer gun, and these parameters for optimization were studied for three different trajectories. 

Optimization using the proposed algorithm is based on the search for an optimized trajectory within the manipulator workspace, always maintaining the orientation of the final effector.

The method was validated by simulating the manipulator displacement in its original and optimized trajectories. The obtained results demonstrate that the algorithm serves to optimize trajectories given its ease of implementation and searches only for values in its objective function.

As can be seen, it is possible to optimize the trajectory time, electric energy and the performance of the manipulator. Likewise, this article presents a general method where, given a trajectory and known manipulator specifications, it is possible to optimize them. 

The way in which the problem has been raised allows two other parameters in the cost function to be easily optimized, with results that provide significant economic savings. In addition, due to the modularity of the method used, the optimization method could also be changed. As a disadvantage of the method, it should be noted that it is necessary to know the dynamic parameters of the manipulator for the correct calculation. On the other hand, this method can be used as a tool for checking dynamic parameters in the design phase of the manipulator. 

An application in C language has been created to extend the developed algorithm to other robots/trajectories. Robot parameters can be entered as inputs. In addition, the desired weight can be assigned to each of the optimized factors (manipulability and electric energy). This paper can be considered a starting point for future research on manipulator control, the identification of manipulator parameters, data validation or the comparison of different optimization methods, as well as the digitalization of movements and their testing through virtual commissioning.

## Figures and Tables

**Table 2:** Comparison of results with different weights for Trajectory 1.

	Weights		Iteration	Energy (J)	Manipulability
	Energy	Manipulability			
Trajectory 1	0.01	0.99	49	9.63	0.11
	0.05	0.95	53	9.11	0.112
	0.5	0.5	46	9.1	0.111
	0.95	0.05	51	8.99	0.114
	0.99	0.01	80	8.66	0.109

**Table 3:** Percentages of the optimization of trajectories of sealer application for Trajectory 1, Trajectory 2 and Trajectory 3.

		Trajectory 1	Trajectory 2	Trajectory 3
Original trajectory	Trajectory points	71	104	135
	Trajectory time (s)	63.2	77.5	89.5
	Average manipulability	0.05	0.1	0.08
	Average consumption (kWh × 10^−6^)	3.51	18.5	2.7
Optimized trajectory	Trajectory time (s)	56.4	74.2	86.2
	Average manipulability	0.121	0.119	0.112
	Average consumption (kWh × 10^−6^)	1.03	14.8	1.46
	Iteration	30	27	38
Percentage of optimized time (%)		10.75	4.25	3.68
Percentage of optimized manipulability (%)		58.67	15.96	28.57
Percentage of optimized electric energy (%)		70.65	20	45.92

**Table 4:** Economic savings with the algorithm’s implementation.

		Trajectory 1	Trajectory 2	Trajectory 3
Trajectory time (s)	Initial trajectory	63.2	77.5	89.5
	Optimized trajectory	56.4	74.2	86.2
	Δt per day	6.8	3.3	3.3
Production/day (bodies)	Initial trajectory	1186	967	838
	Optimized trajectory	1329	1010	870
	ΔProduction per day	143	43	32
Economic savings (EUR/month)		67,782	20,382	15,168

**Table 5:** Relevant e-KPIs with the algorithm’s implementation.

		Trajectory 1	Trajectory 2	Trajectory 3
Original trajectory	Energy intensity (kWh × 10^−6^/vehicle)	0.0029	0.0191	0.0032
	Basic electricity charge indicator (kWh × 10^−6^)	0.75	0.94	0.68
Optimized trajectory	Energy intensity (kWh × 10^−6^/vehicle)	7.75 × 10^−4^	0.0146	0.0016
	Basic electricity charge indicator (kWh × 10^−6^)	0.34	0.93	0.48

## Data Availability

Not applicable.
